# Phylogeny and polymorphism in the E6 and E7 of human papillomavirus: alpha-9 (HPV16, 31, 33, 52, 58), alpha-5 (HPV51), alpha-6 (HPV53, 66), alpha-7 (HPV18, 39, 59, 68) and alpha-10 (HPV6, 44) in women from Shanghai

**DOI:** 10.1186/s13027-019-0250-9

**Published:** 2019-11-21

**Authors:** Junwei Zhao, Qin Zhan, Junhan Guo, Min Liu, Yetian Ruan, Tailin Zhu, Lingfei Han, Fang Li

**Affiliations:** 1 Department of Obstetrics and Gynecology, East Hospital, Tongji University School of Medicine, 150 Jimo Rd, Shanghai, 200120 People’s Republic of China; 2 Department of Gynecology, Shanghai First Maternity and Infant Hospital, Tongji University School of Medicine, Shanghai, 201204 China; 3 School of Physics HH Wills Physics Laboratory, University of Bristol, Bristol, UK

**Keywords:** Human papillomavirus, Cervical intraepithelial neoplasia, *E6* and *E7* gene, Genetic variations, Polymorphism analysis

## Abstract

**Background:**

Persistent infection with human papillomaviruses (HPVs) has been associated with cervical intraepithelial neoplasia (CIN) and cervical cancer. However, why only a fraction of HPV cases progress to cancer is still unclear.

**Methods:**

We focused on the heterogeneity, classification, evolution and dispersal of variants for 14 common HPV types in 262 HPV-positive patients with cervical lesions. The *E6* and *E7* genes of HPV were sequenced and compared with the HPV reference for sequence analysis. Phylogenetic trees were constructed using the neighbour-joining tree method with MEGA 7.0.

**Results:**

In this study, 233 *E6* and 212 *E7* sequences were successfully amplified by PCR, and these sequences were divided into 5 species groups: alpha-9 (HPV16, 31, 33, 52, 58), alpha-5 (HPV51), alpha-6 (HPV53, 66), alpha-7 (HPV18, 39, 59, 68) and alpha-10 (HPV6, 44). The incidence of high-grade squamous intraepithelial lesion (HSIL) in patients infected with alpha-9 HPV was significantly increased compared with other groups (*P* < 0.0001), especially HPV16 (*P* < 0.0001). Strikingly, *E7* had significantly fewer nonsynonymous variants in the HSIL compared to <HSIL groups (*P* = 3.17× 10^− 4^). The A388C (K93 N) variation in HPV58 *E6* can significantly reduce the risk of HSIL (*P* = 0.015). However, T7220G (D32E) variation in HPV16 *E6* and A7689G (N29S) in HPV16 *E7* increased the incidence of HSIL compared to the <HSIL group (*P* = 0.036 and 0.022).

**Conclusions:**

Strict conservation of E7 is important for HPV carcinogenicity, especially N29 of HPV16. The findings in this work provide preventative/therapeutic interventions for HPV infections and CIN.

## Background

Currently, over 200 types of HPV have been fully characterized, of which the great majority clusters phylogenetically within three genera of the Papillomaviridae family: alpha (α), beta (β), and gamma (γ) [1]. The α genus contains HPV types that infect mostly mucosal and genital regions, including 65 papillomavirus types from humans, and this group of viruses constitutes 14 species groups [2]. Persistent HPV infections are considered the material cause of cervical cancer, where greater than 99% of cervical cancer lesions contain HPV DNA [3]. At least 3 ancestral papillomaviruses are responsible for the current heterogeneous groups of genital HPV genomes, including low-risk (LR)1 (α1, 8, 10 and 13), LR2 (α2, 3, 4 and 14) and high-risk (HR) (α5, 6, 7, 9 and 11) [2].

However, why only a small proportion of HPV infections progressed to precancer and cancer is unclear [4]. In addition to the pathogenic heterogeneity of distinct HPV types, previous studies indicate that HPV variants are also associated with different risks of cancer progression. For example, the HPV16 variant has significantly different risks of HPV persistent infection, progression to cervical intraepithelial neoplasia (CIN) and cervical cancer [5, 6]. Lisa Mirabello observed that compared to the most frequent A1/A2 sublineages, the A4, C, D2 and D3 sublineages conferred a higher hazard of CIN and cervical cancer [7]. The C variant (vs. B variant) of HPV52 was associated with an increased prevalence of cytologically diagnosed and histologically confirmed HSIL or worse lesions [8]. These data indicate that HPV variants have different phenotypic characteristics, including carcinogenicity.

HPV E6 and E7 are the major oncogenes, which are highly expressed in tumours and are related to inducing cellular immortalization, transformation, and carcinogenesis through protein–protein interactions with tumour suppressor proteins [9]. For example, E6 binds the conserved LxxLL consensus sequences of the ubiquitin ligase E6-associated protein (E6-AP), which works as a connecting bridge between E6 and p53, leading to its subsequent degradation [10]. Similarly, E7 targets and promotes the inactivation of RB1, thus inducing cell-cycle progression through activation of E2F-driven transcription [11].

In this study, we focused on the phylogeny and polymorphism of *E6* and *E7* gene variants for 14 common HPV types (HPV16, 31, 33, 52, 58, 51, 53, 66, 18, 39, 59, 68,6, 44) in Shanghai women with cervical lesions. This comprehensive analysis will help us understand the clinical and biological role of sequence variation.

## Materials and methods

### Study population

In total, 262 HPV-positive patients (mean age 38.34 ± 10.52 years, 21–78) with histopathologically confirmed cervical lesions, including 92 nonneoplastic, 69 low-grade squamous intraepithelial lesion (LSIL) and 101 high-grade squamous intraepithelial lesion (HSIL), were recruited from the Cervical Disease Centre at the Shanghai First Maternity and Infant Hospital, Tongji University School of Medicine in Shanghai, China. Histopathological findings are divided into certain groups as nonneoplastic (chronic cervicitis and inflammation-related regenerative changes), LSIL (CIN I/mild dysplasia), HSIL (CIN II and CIN III/moderate and severe dysplasia) and invasive carcinoma. CIN I refers to mildly atypical cellular changes in the lower third of the epithelium, CIN II refers to moderately atypical cellular changes confined to the basal two-thirds of the epithelium (formerly called moderate dysplasia) with preservation of epithelial maturation. CIN III refers to severely atypical cellular changes encompassing greater than two-thirds of the epithelial thickness and includes full-thickness lesions (previous terms were severe dysplasia or carcinoma in situ).

The criteria for the inclusion of patients enrolled into their current study: HPV single infection; Histopathologically confirmed by Colposcopy biopsy. The exclusion: Co-infected with different HPV types; Not histopathologically confirmed; the patients with vaginitis or other bacterial/virus infection.

### Genomic DNA isolation and HPV typing

DNA from exfoliated cervical cells was extracted using the TIANamp Genomic DNA Kit (No: 3304–9) according to the manufacturer’s instructions. HPV genotyping was conducted using an HPV GenoArray Test Kit (HybriBio Ltd).

### Amplification and sequencing

After HPV testing, the remaining DNA samples were stored at − 80 °C and used to amplify *E6* and *E7* using specific primers (Table 1). Subsequently, PCR products excised from 1.5% agarose gel were sequenced bidirectionally by SAIYIN Gene Biotechnology Company, Shanghai, China.

**Table 1:** Primers used for the molecular characterization of fourteen human papillomavirus *E6* and *E7* genes

HPV genotype	Reference sequence ID	Gene	Direction	Sequence 5′-3’	Primer position	Product size, bp	Annealing Temperature, °C
6	KU298876.1	*E6*, *E7*	Forward	AGGGACCGAAAACGGTTCAA	32	1079	58
			Reverse	CTAACATATGGACTACCTAAAT	1110		
16	NC_001526	*E6*	Forward	ACCGTTTTGGGTTACACATTTAC	6996	700	60
			Reverse	CTGTCATTTAATTGCTCATAACAGTAGA	7695		
		*E7*	Forward	CATTAGAACAGCAATACAACAAACC	7405	579	60
			Reverse	TCCACTACAGCCTCTACATAAAACC	7983		
18	NC_001526	*E6*, *E7*	Forward	CATGTCCAACATTCTGTCTACCC	7751	1064	58
			Reverse	TTACAACCCGTGCCCTCC	957		
31	J04353.1	*E6*, *E7*	Forward	AGTAGGGAGTGACCGAAAGTGG	27	959	58
			Reverse	CACTACTGTCTTCATTTTCGTCCTC	985		
33	M12732.1	*E6*, *E7*	Forward	AACTATGCCTTGTAAAAGTGAGTCAC	7813	1116	58
			Reverse	TAAATCCGTGCCACTGTCATC	1015		
39	M62849.1	*E6*, *E7*	Forward	AAGGGAGTAACCGAAAACGG	34	1096	58
			Reverse	CCTGTGCTGTCTCACGCTCT	1129		
44	U31788.1	*E6*, *E7*	Forward	ATCGGTTGACACACACCCTG	7796	1083	58
			Reverse	CATCCGCCTCCTGTCGTTTAACAA	1045		
51	KU298901.1	*E6*, *E7*	Forward	ACTAGGGTGTAACCGAAAAGGG	17	965	58
			Reverse	TCATCCTCATCATCCGAAACAT	981		
52	NC_001592.1	*E6*	Forward	ACCGTACCCACAACCACTTTT	7929	738	58
			Reverse	TTGTGGCTTGTTCTGCTTGTC	706		
		*E7*	Forward	AACGCCATTATGTCCTGAAGAA	422	554	58
			Reverse	CATCCTCGTCCTCTGAAATGTTAT	975		
53	GQ472849.1	*E6*, *E7*	Forward	AGACAGGGAGTAACCGAAATAGG	24	988	58
			Reverse	GCTTTCCTCGTCTGTTTCATCTT	1011		
58	D90400	*E6*	Forward	CGTTTTGGGTCACATTGTTCA	7782	702	58
			Reverse	CATAATTGCTCATAGCAGAATAGGTC	659		
		*E7*	Forward	TTCGCTATATGGAGACACATTAGAA	352	613	58
			Reverse	TTCTTCGTTCTATTACCGCTTCTA	964		
59	X77858.1	*E6*, *E7*	Forward	AAGCAACCGAAAAAGGTCGG	7805	1128	58
			Reverse	TGTGGTATCATCAATAAAATCTACC	1036		
66	U31794.1	*E6*, *E7*	Forward	TTGGGAGTAACCGAAATGGG	27	992	58
			Reverse	CATTCTCCTCCTCGCTTTCAT	1018		
68	DQ080079	*E6*, *E7*	Forward	CCGAAAAAGGTTGGGCACAC	7682	1098	58
			Reverse	TGAACCTGTATCTGTTGCGTT	958		

*HPV* Human papillomavirus

### Phylogenetic tree analysis and sequence analysis

The neighbour-joining phylogenetic tree of the HPVs was constructed by *MEGA 7.0* using the maximum composite likelihood estimate [12]. To construct distinct phylogenetic branches, the reference HPV sequences were obtained from the GenBank database. The phylogenetic trees were visualised in FigTree v1.4.3 and online Evolview [13, 14].

The sequences were subsequently analysed by NCBI Blast, and all unique sequences were compared pairwise using the *ClustalW* tool of *MEGA 7.0*. The nucleotide positions of HPV were numbered on the basis of the reference sequence KU298876.1 (HPV6), NC_001526 (HPV16), NC_001526 (HPV18), J04353.1 (HPV31), M12732.1 (HPV33), M62849.1 (HPV39), U31788.1 (HPV44), KU298901.1 (HPV51), NC_001592.1 (HPV52), GQ472849.1 (HPV53), D90400 (HPV58), X77858.1 (HPV59), U31794.1 (HPV66), and DQ080079 (HPV68).

### Statistical analysis

Fisher’s exact test was chosen for statistical analysis. *P* < 0.05 was used as the threshold to indicate statistical significance. All the *P* values in the present study were two-sided. The power calculation was performed by G*power software [15].

## Results

**Fig. 1:**
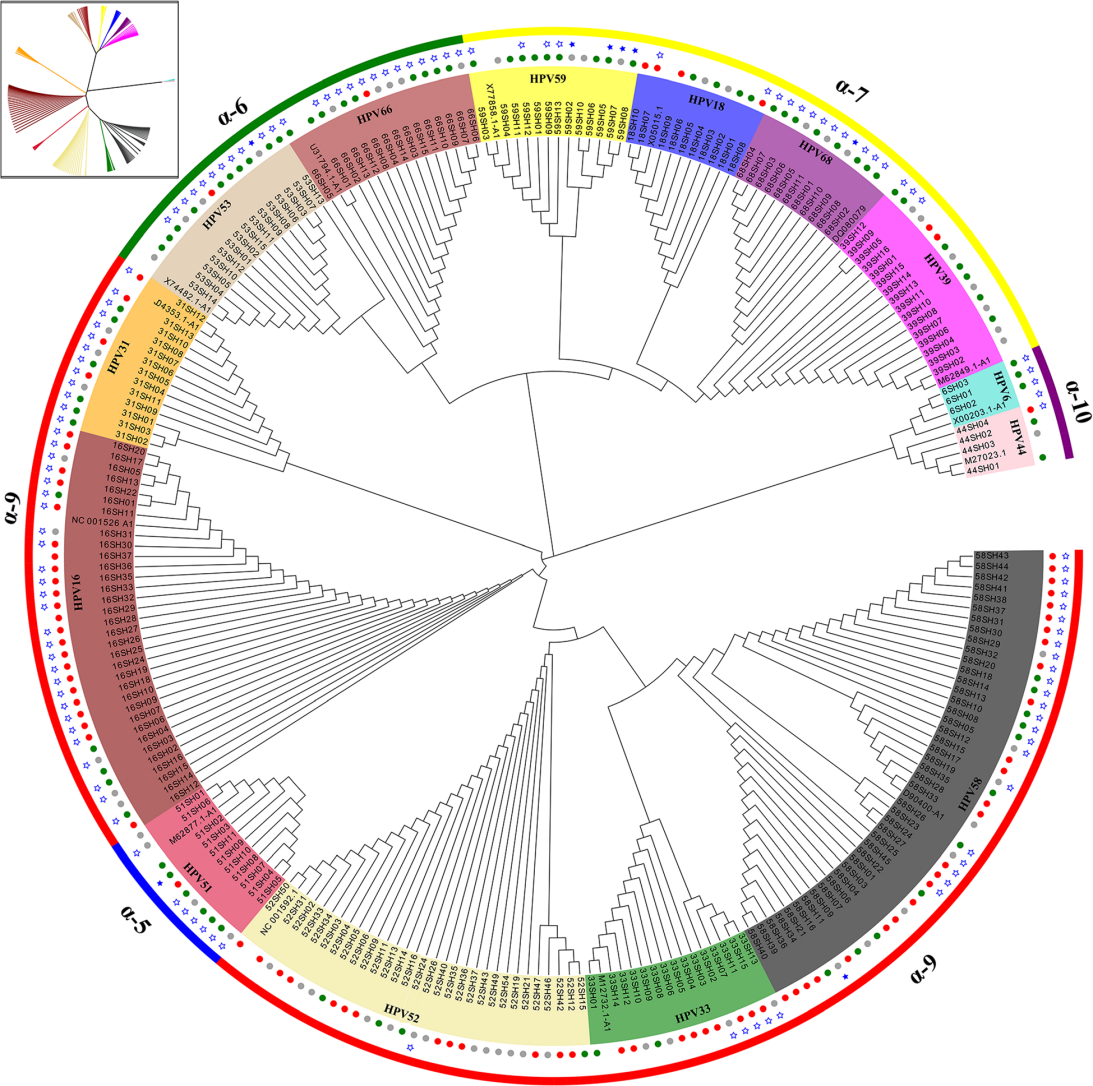
Phylogenetic tree of Alphapapillomavirus based 233 nucleotide sequence alignments of HPV *E6*. The maximum likelihood tree was constructed using MEGA7.0. Phylogenetic trees were visualised in FigTree v1.4.3 and Evolview. These sequences were divided into 5 species groups (alpha-5, alpha-6, alpha-7, alpha-9, alpha-10), of which alpha-10 was a low-risk (LR) clade. Green, grey and red circle represent cervicitis, low-grade squamous intraepithelial lesion, high-grade squamous intraepithelial lesion, respectively; the star represents nonsynonymous mutation, and blue stars are insertion/deletions

**Fig. 2:**
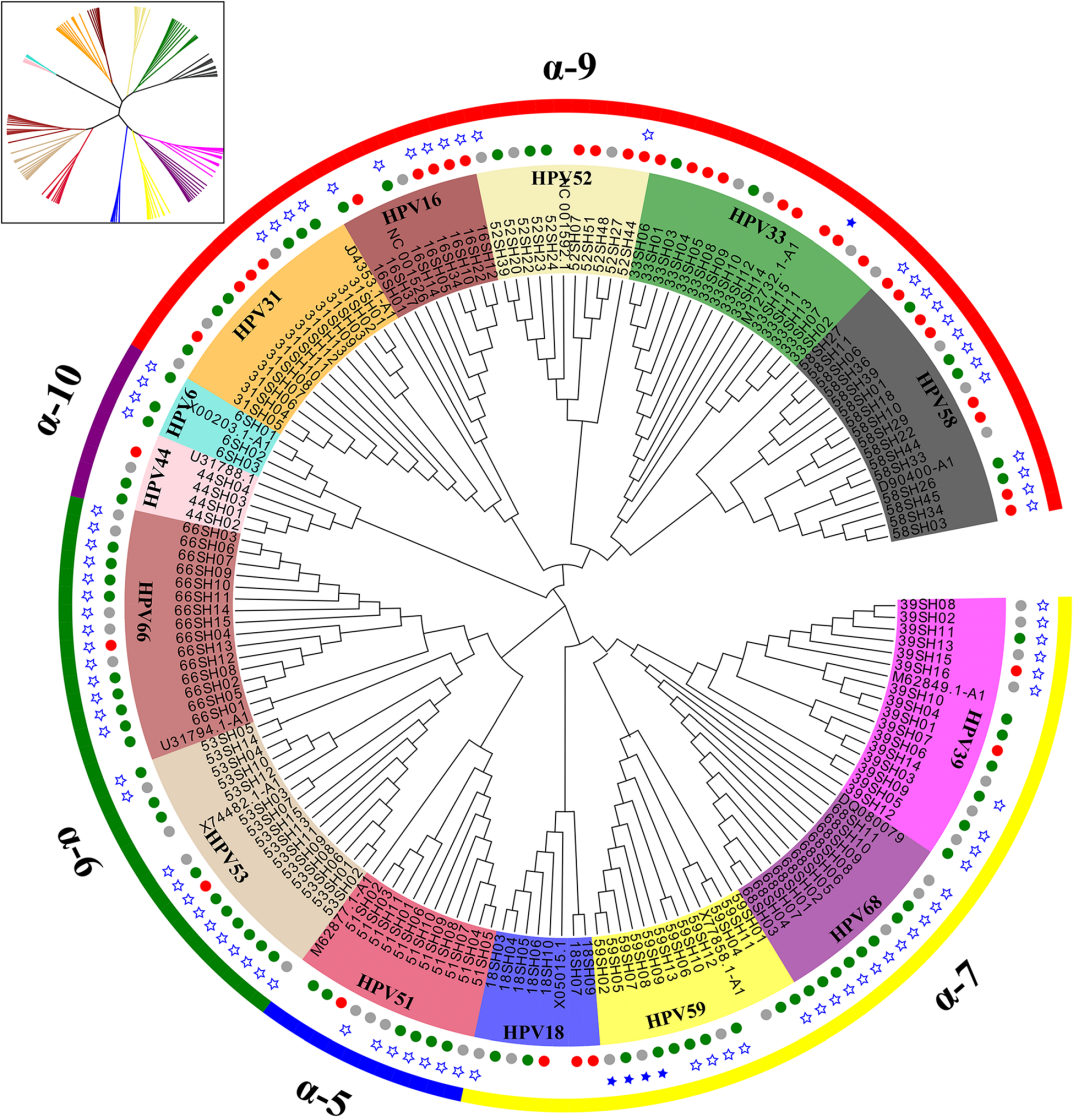
Phylogenetic tree of Alphapapillomavirus based 156 nucleotide sequence alignments of HPV *E7*. The maximum likelihood tree was constructed using MEGA7.0. Phylogenetic trees were visualised in FigTree v1.4.3 and Evolview. These sequences were divided into 5 species groups (alpha-5, alpha-6, alpha-7, alpha-9, alpha-10), of which alpha-10 was a low-risk (LR) clade. Green, grey and red circle represent cervicitis, low-grade squamous intraepithelial lesion, high-grade squamous intraepithelial lesion, respectively; the star represents nonsynonymous mutations, and blue stars are insertion/deletions

In this study, total DNA was extracted from exfoliated cervical cell samples from 262 HPV-positive patients. In total, 233 *E6* and 212 *E7* sequences were successfully amplified by PCR. Based on the reference sequences, we confirmed that these sequences were divided into 14 types of HPV (16, 31, 33, 52, 58,51,53, 66,18, 39, 59, 68, 6, 44) and 5 species groups (alpha-5, alpha-6, alpha-7, alpha-9, alpha-10) using phylogenetic tree analysis, where alpha-10 was a low-risk (LR) clade (Fig. 1 and Fig. 2).

Table 2 shows the distribution of the sublineage-specific infections for individual types in cervicitis, LSIL and HSIL groups. The incidence of HSIL was significantly increased in patients infected with alpha-9 HPV compared with other species (*P* < 0.0001), especially HPV16 (*P* < 0.0001). There was no statistically significant difference in the severity of CIN for all types of lineages. For HPV16, 37.5% HPV16 A1–3 sub-lineage caused HSIL, as well as 75.9% A4. Among 54 determinable samples of HPV 52, the A, B and C variants were found in 1 (1.85%), 52 (96.3%) and 1 (1.85%) samples, respectively, and lineage B was the most common. Among 45 determinable samples of HPV 58, sublineages A1, A2 and A3 variants were found in 57.8, 22.2 and 17.8% of all HPV58 samples, respectively. The nonprototype-like variant (sublineage B1) of HPV58 was rare in our study. A2 (69.23%, 9/13) and A1 (66.67%, 10/15) were common sublineages for HPV31 and HPV33, respectively.

**Table 2:** Distribution of lineage-specific human papillomavirus infections in samples from Shanghai

		Age^#^	Lineage or	Women	pathologic diagnosis			
Genus	Type	(Mean±SD)	sublineage	N	IF	LSIL	HSIL	P^*^ Value
α-9				**164**	**34**	**39**	**91**	**<0.0001**^a^
	16	34.88 ± 6.15	A1-3	8	3	2	3	0.083
		35.10 ± 6.83	A4	29	2	5	22	
	31	33.33 ± 8.41	A2	9	3	2	4	0.108
			B2	1	0	1	0	
		31.67 ± 5.51	C2	3	3	0	0	
	33	38.90 ± 11.57	A1	10	2	2	6	0.417
			A2	1	0	1	0	
		39.75 ± 8.18	A3	4	0	1	3	
	52		A	1	0	0	1	1
		38.48 ± 11.83	B	52	10	17	25	
			C	1	0	0	1	
	58	40.19 ± 9.55	A1	26	7	4	15	0.862
		40.30 ± 7.54	A2	10	1	3	6	
		44.25 ± 14.44	A3	8	3	1	4	
			B1	1	0	0	1	
α-5				**11**	**6**	**4**	**1**	
	51		A1	1	0	1	0	0.532
		36.50 ± 7.94	A2	4	2	1	1	
		39.83 ± 14.68	A4	6	4	2	0	
α-6				**30**	**19**	**9**	**1**	
	53	34.00 ± 5.66	A1	2	1	1	0	0.588
		42.50±12.02	B1	2	1	1	0	
		33.33±10.41	C1	3	2	1	0	
		31.00 ± 7.07	D1	2	1	1	0	
		36.00 ± 13.36	D3	6	5	0	1	
	66		A1	1	1	0	0	0.552
		45.17 ± 13.53	B1	6	4	1	1	
		42.50 ± 13.18	B2	8	4	4	0	
α-7				**50**	**28**	**16**	**6**	
	18	34.50 ± 10.42	A1	10	4	2	4	0.4
	39	39.08 ± 10.49	A1	13	5	6	2	1
		43.00 ± 22.63	A2	2	1	1	0	
			B1	1	1	0	0	
	5	33.50 ± 8.96	A1	4	3	1	0	0.664
		36.00±2.65	A3	3	3	0	0	
		36.50±6.36	B1	2	1	1	0	
		51.25 ± 5.00	B1-2	4	2	2	0	
	68	45.00 ± 10.90	C1	9	6	3	0	1
		35.00 ± 8.49	C2	2	2	0	0	
α-10				**7**	**5**	**1**	**1**	
	6	34.33 ± 12.06	B1	3	3	0	0	
	44	32.50 ± 4.04		4	2	1	1	
Total				262	92	69	101	<0.0001^b^

*IF* Cervicitis, *LSIL* Low-grade squamous intraepithelial lesion, *HSIL*, High-grade squamous intraepithelial lesion; ^a^Comparison of types within α-9 group; ^b^Comparison between Genus; ^*^P values remain significant after Bonferroni adjustment for multiple tests. ^#^*P*<0.05 using analysis of variance. The boldface entries indicate the distribution of α-5, α-6, α-7, α-9 and α-10 HPV infection in different populations (IF, LSIL and HSIL group)

**Fig. 3:**
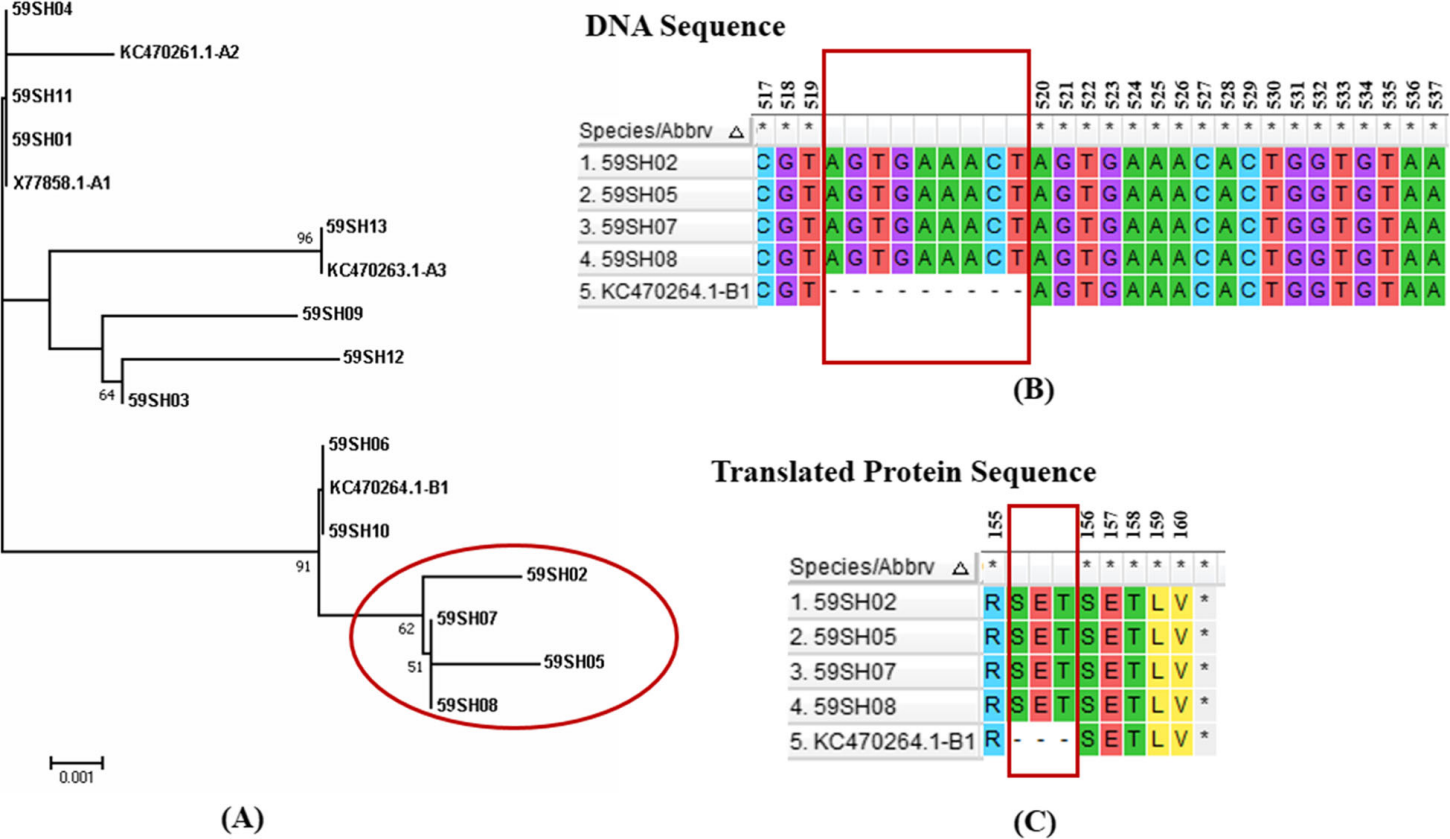
Phylogenetic tree and schematic representation of 4 novel HPV59 E6 variants. **a** Phylogenetic tree of the HPV59 variants based on *E6* sequences. **b** An insertion (AGTGAAACT) at nucleotide sites 519 in 4 variants. **c** The insertion sequence was translated into SET. Four variants are marked by red circles

Interestingly, we observed that one variant represented four out of 13 HPV59-positive samples that appeared to form a new candidate, sublineage B1–2 (Fig. 3a). A 9-base sequence (AGTGAAACT) was inserted after position 519 of the *E6* sequence, and 9 inserted bases were translated into 3 amino acids SET (Fig. 3b and c). These diagnostic SNPs were unique to the B1–2 sublineage.

Nonsynonymous mutations for the *E6* and *E7* genes within all types of HPV were evaluated. The A burden test was used to determine if the variant distribution was different between the IF, LSIL and HSIL groups by viral region (Table 3, Fig. 1, and Fig. 2). Despite nearly equal numbers of E6 and E7 sequences among three groups (IF, 159; LSIL, 121; HSIL, 165), the IF group overall had a significantly higher number of variants compared to the LSIL and HSIL groups (*P* = 3.83× 10^− 4^). Strikingly, the *E7* gene had significantly fewer nonsynonymous variants in the HSIL compared to LSIL and IF groups (*P* = 3.17× 10^− 4^).

**Table 3:** Rare variant burden analysis for nonsynonymous variants within all types of HPV for the cervicitis, LSIL and HSIL groups

		N Controls			N LSIL			N HSIL		
Viral	Number	with	% of	Number	with	% of	Number	with	% of	
Gene	IF	Variants	Controls	LSIL	Variants	Controls	HSIL	Variants	Controls	*P* Value
E6	86	55	64.0	63	32	50.8	84	45	53.6	0.215
E7	73	53	72.6	58	31	53.4	81	32	39.5	3.17 × 10^–4*^
Total	159	108	67.9	121	63	52.1	165	77	46.7	3.83 × 10^–4*^

*HPV* Human papillomavirus; *IF* cervicitis; *LSIL* low-grade squamous intraepithelial lesion; *HSIL* high-grade squamous intraepithelial lesion;^*^P values remain significant after Bonferroni adjustment for multiple tests

Moreover, we confirmed that the incidence of HSIL in patients infected with the alpha-9 HPV group was significantly increased compared with the other groups (*P* < 0.0001). We then further analysed nonsynonymous mutations of the alpha-9 HPV (HPV16, 31, 33, 52, 58) *E6* and *E7* genes in the HSIL case and control groups (Table 4). In the case group, 13 variations were observed in the *E6* gene, and 19 mutations were observed in the *E7* gene. In the control group, 17 and 14 variations were found in the *E6* and *E7* genes, respectively. For HPV16, the distribution of T7220G (D32E) variation in *E6* and A7689G (N29S) in *E7* showed a different trend between the case group and control group (*P* = 0.036 and 0.022) (Table 4), power (1-β) 0.562 and 0.629. For HPV58, A388C (K93 N) variation can significantly reduce the risk of HSIL and was a protective factor (*P* = 0.015), power (1-β) 0.624. In the remaining three types of alpha-9 HPV, no significant differences in the distribution of other variations between the case group and the control group were found. In addition, we performed co-variation analysis of five HPVs *E6* and *E7* genes in the α-9 group. But there was no significant correlation between E6 and E7 covariation and cervical lesions (Additional file 1: Table S1).

**Table 4:** HPV *E6*/*E7* gene variations and amino acid substitutions in the case and control groups

HPV	Genome	Amino	Case^b^		Control^c^		*P* value^*^
	position^a^	acid^a^	Mutation	Frequency(%)	Mutation	Frequency (%)	
HPV16 *E6* case (*n*=23) control (*n*=10)	T7179G	L19V	1	4.3	0	0.0	1.000
	T7220G	D32E	20	87.0	5	50.0	**0.036**
	C7377T	H85Y	0	0.0	1	10.0	0.303
	G7384C	C87S	2	8.7	0	0.0	1.000
	A7404T	T94S	3	13.0	1	10.0	1.000
	A7484C	E120D	1	4.3	1	10.0	0.521
*E7* case (*n*=21) control (*n*=10)	A7688C	N29H	0	0.0	1	10.0	0.323
	A7689G	N29S	19	90.5	5	50.0	**0.022**
	C7832T	R77C	1	4.8	1	10.0	1.000
HPV31 *E6* case (*n*=4) control (*n*=9)	C285T	H60Y	0	0.0	3	33.3	0.497
	A297G	T64A	0	0.0	1	11.1	1.000
	A475G	K123R	0	0.0	1	11.1	1.000
	C520T	A138V	0	0.0	4	44.4	0.228
*E7* case (*n*=4) control (*n*=9)	C626T	H23K	4	100.0	6	66.7	0.497
	G695A	E46K	0	0.0	4	44.4	0.228
	A743G	K62E	4	100.0	9	100.0	—
HPV33 *E6* case (*n*=9) control (*n*=6)	A213C	K35N	3	33.3	2	33.3	1.000
	A364C	N86H	3	33.3	2	33.3	1.000
	A387C	K93N	3	33.3	1	16.7	0.604
	A446G	Q113R	3	33.3	1	16.7	0.604
*E7* case (*n*=9) control (*n*=6)	A834G	N88D	1	11.1	0	0.0	1.000
	C850A	T93N	1	11.1	0	0.0	1.000
	A862T	Q97L	3	33.3	2	33.3	1.000
HPV52 *E6* case (*n*=13) control (*n*=17)	G108C	E3Q	0	0.0	1	5.9	1.000
*E7* case (*n*=27) control (*n*=26)	C624G	C24W	0	0.0	1	3.8	0.491
	C662T	T37I	1	3.7	0	0.0	1.000
	G707A	S52D	1	3.7	0	0.0	1.000
	T727G	Y59D	1	3.7	0	0.0	1.000
	C733T	H61Y	1	3.7	0	0.0	1.000
	G742A	D64N	1	3.7	0	0.0	1.000
	T848G	L99R	1	3.7	0	0.0	1.000
HPV58 *E6* case (*n*=25) control (*n*=18)	G203C	E32Q	3	12.0	1	5.6	0.628
	C228T	S40F	1	4.0	0	0.0	1.000
	C367A	D86E	2	8.0	2	11.1	1.000
	A388C	K93N	1	4.0	6	33.3	**0.015**
	A544T	K145S	0	0.0	1	5.6	0.419
*E7* case (*n*=12) control (*n*=9)	C632T	T20I	3	25.0	3	33.3	1.000
	G694A	G41R	3	25.0	2	22.2	1.000
	C755A	T61N	0	0.0	1	11.1	0.429
	G760A	G63S	4	33.3	2	22.2	0.659
	G761A	G63D	3	25.0	2	22.2	1.000
	A793G	T74A	1	8.3	0	0.0	1.000
	C801A	D76E	1	8.3	0	0.0	1.000
	T803C	V77A	0	0.0	3	33.3	0.063

*HPV* Human papillomavirus; ^a^The reference HPV16/31/33/52/58 E6/E7 gene sequence was NC_001526, J04353.1, M12732.1, NC_001592.1, and D90400. ^b^HSIL group, ^c^LSIL and IF group. ^*^ Fisher's exact test *P* value, and the bold numbers refer *P* value less than 0.05

## Discussion

Persistent infection with HPV is the most important risk factor for cervical cancer [16]. According to their oncogenic potential, HPV types are divided into high-risk HPV types (16, 18, 31, 33, 35, 39, 45, 51, 52, and 58) associated with cervical cancers, and low-risk types (6, 11, 40, 42, 43, 44, and 54) associated with genital warts [17]. The E6 and E7 oncoproteins of HPV contribute to oncogenesis by associating with the tumour suppressor proteins p53 and pRb, respectively [18]. In this report, we describe the E6 and E7 genes of 14 conventional HPV species (HPV16, 31, 33, 52, 58,51,53, 66, 18, 39, 59, 68,6, 44) in Shanghai women with cervical lesions. This work provides basic information and reference variant sequences for future investigation of viral-host evolution and viral pathogenesis.

In this study, the α-9 (HPV16, 31, 33, 52, 58), α-5 (HPV51), α-6 (HPV53, 66), α-7 (HPV18, 39, 59, 68) and α-10 (HPV6, 44) were were detected and analyzed. 79.26% of α-9 HPV infection caused CIN confirmed histopathologically, 55.49% of which were HSIL. HPV16 A4, HPV31 A2, HPV33 A1, HPV52 B and HPV58 A1 were the most common sublineages in the α-9 HPV group. In China, the A1-A3 sublineage of HPV16 was predominant in northeast China [19], and A4 was common in central and south China [20, 21]. Globally, the risk of cervical cancer caused by the A3, A4 and D sublineages was significantly higher compared with HPV16 A1 [22]. In our study, HPV 31/33/52/58 had variant lineages similar to those reported by previous studies, and sublineages associated with CIN and/or cervical cancer were HPV52 C and HPV33 A1 [8, 23–26]. We should improve the screening of cervical cancer based on HPV pathogenic sub-lineages in different regions. This also reduces the rate of colposcopy biopsy, which can reduce the burden on patients and reduce the waste of medical resources. Simple infections of HPV16 carcinogenic subtypes or low-grade lesions caused by them should be intervened as early as possible rather than just follow-up. However, the sample size should be expanded to further confirm our research results.

The genome variations in humans and HPV may influence any stage of HPV infection by inducing cervical cancer [27]. For E6, the T7220G (D32E) variation in HPV16 E6 was a risk factor that increased the incidence of HSIL, whereas A388C(K93 N) variation in HPV58 E6 significantly reduced the risk of HSIL. Previous studies have shown that the susceptibility to cervical disease is increased by the specific protein interaction HPV16 E6 (L83 V)-p53 (Arg-72, [28]. Moreover, the gene variant T350G of HPV-16 was found to display more efficient degradation of Bax and binding to the E6 binding protein [29]. We found that E7 was highly conserved in the HSIL group compared to the <HSIL group, and A7689G (N29S) in *E7* significantly increased the risk of HSIL. While the HPV16 A4 sublineage (*P* < 0.0001) and HPV16 E7 29S (*P* = 0.0002) rarely occurred in cancer patients compared to women with cervicitis in Vietnam [30]. HPV16 E7 S63F was significantly different between the case and control groups (*P* = 4.861 × 10^− 10^) in a Han Chinese population [31]. The T20I/G63S substitutions in HPV16 A3 E7 significantly increased the risk for HSIL in Taizhou area, China [32]. In one word, HPV sub-lineage and variation dispersal was population-specific, and we should develop different screening and treatment schemes according to the distribution of HPV variation in different regions. Due to the limitation of sample capacity, we should increase the sample size to confirm the role and mechanism of these mutations in the development of cervical cancer in Shanghai area or south China.

In current study, the E7 gene had significantly fewer nonsynonymous variants in the HSIL compared to LSIL and IF groups (*P* = 3.17× 10–4). Lisa Mirabello et al. confirmed hypovariation in that E7 had significantly fewer, rare non-silent genetic variants in cancers (*P* = 6.13× 10^− 5^) compared to E6 [33]. Previous studies have reported that the HPV16 E7 protein leading to cervical cancer is virtually invariant, and E7 displayed a fully conserved sequence [34, 35]. In summary, E7 variation greatly decreases the risk of CIN and invasive cancer.

## Conclusions

In this study, we focused on the phylogeny and polymorphism of 14 HPV variants based on the *E6* and *E7* genes. In addition, we also found that the *E7* gene lacked significant genetic variation in CIN, and which was strict conservation in the HSIL. This comprehensive analysis will help us understand the clinical and biological effects of sequence changes and provide preventative/therapeutic interventions for HPV-related CIN and cervical cancer.

## Supplementary information

**Additional file 1 Table S1.** Co-variations analysis of α-9 HPV E6 and E7 gene in the case and control groups.

## Data Availability

The datasets used and/or analysed during the current study are available from the corresponding author on reasonable request.

## Abbreviations

CIN: Cervical intraepithelial neoplasia
HPV: Human papillomavirus
HSIL: High-grade squamous intraepithelial lesion
LSIL: Low-grade squamous intraepithelial lesion
