# Supplementary material for: Phylogeny and polymorphism in the E6 and E7 of human papillomavirus: alpha-9 (HPV16, 31, 33, 52, 58), alpha-5 (HPV51), alpha-6 (HPV53, 66), alpha-7 (HPV18, 39, 59, 68) and alpha-10 (HPV6, 44) in women from Shanghai
Source: Infect Agent Cancer. 2019 Nov 21;14:38. doi: 10.1186/s13027-019-0250-9 (PMC6873513; doi:10.1186/s13027-019-0250-9)
Supplement: Supplementary file 1 — Additional file 1 Table S1. Co-variations analysis of α-9 HPV E6 and E7 gene in the case and control groups. [file 13027_2019_250_MOESM1_ESM.docx]

**Table S1. Co-variations analysis of α-9 HPV E6 and E7 gene in the case and control groups.**

| **HPV** | **E6** | | | | | | **E7** | | | | | | | | **Control^c^** | **Case^b^** | ***P*^*^Value** |
| --- | --- | --- | --- | --- | --- | --- | --- | --- | --- | --- | --- | --- | --- | --- | --- | --- | --- |
| **HPV 16** | **7688^a^** | **7689** | **7832** |  |  | **7179** | | **7220** | **7377** | **7384** | **7404** | **7484** |  |  | 18 | 9 | 0.052 |
|  | **A** | **A** | **C** |  |  | **T** | | **T** | **C** | **G** | **A** | **A** |  |  |  |  |  |
|  | — | — | — |  |  | — | | — | — | — | — | — |  |  | 1 | 1 |  |
|  | — | — | — |  |  | — | | — | — | — | T | — |  |  | 1 | 0 |  |
|  | — | G | — |  |  | — | | — | — | — | — | — |  |  | 1 | 0 |  |
|  | — | G | — |  |  | — | | G | — | — | — | C |  |  | 0 | 1 |  |
|  | — | G | — |  |  | G | | G | — | — | — | — |  |  | 0 | 1 |  |
|  | — | G | — |  |  | — | | G | — | — | T | — |  |  | 0 | 1 |  |
|  | — | G | — |  |  | — | | G | — | — | — | — |  |  | 14 | 3 |  |
|  | — | — | T |  |  | — | | — | — | — | — | — |  |  | 1 | 1 |  |
|  | C | — | — |  |  | — | | — | T | — | — | C |  |  | 0 | 1 |  |
| **HPV 31** | **285** | **297** | **475** | **520** |  | **626** | | **695** | **743** |  |  |  |  |  | 8 | 5 | 0.648 |
|  | **C** | **A** | **A** | **C** |  | **C** | | **G** | **A** |  |  |  |  |  |  |  |  |
|  | T | — | — | T |  | — | | A | G |  |  |  |  |  | 3 | 0 |  |
|  | — | G | G | T |  | T | | A | G |  |  |  |  |  | 1 | 0 |  |
|  | — | — | — | — |  | T | | — | G |  |  |  |  |  | 4 | 5 |  |
| **HPV33** | **213** | **364** | **387** | **446** |  | **834** | | **850** | **862** |  |  |  |  |  | 6 | 9 | 0.849 |
|  | **A** | **A** | **A** | **A** |  | **A** | | **C** | **A** |  |  |  |  |  |  |  |  |
|  | — | — | — | — |  | — | | — | — |  |  |  |  |  | 4 | 6 |  |
|  | C | C | — | — |  | — | | — | T |  |  |  |  |  | 1 | 0 |  |
|  | C | C | C | G |  | G | | A | T |  |  |  |  |  | 0 | 1 |  |
|  | C | C | C | G |  | — | | — | T |  |  |  |  |  | 1 | 2 |  |
| **HPV52** | **108** |  |  |  |  | **624** | | **662** | **707** | **727** | **733** | **742** | **848** |  | 16 | 13 |  |
|  | **G** |  |  |  |  | **C** | | **C** | **G** | **T** | **C** | **G** | **T** |  |  |  |  |
|  | C | — | — | — | — | — | | — | — | — | — | — | — |  | 1 | 0 |  |
|  | — | — | — | — | — | — | | — | — | — | — | — | — |  | 15 | 13 |  |
| **HPV58** | **203** | **228** | **367** | **388** | **544** | **632** | | **694** | **755** | **760** | **761** | **793** | **801** | **803** | 9 | 12 | 0.249 |
|  | **G** | **C** | **C** | **A** | **A** | **C** | | **G** | **C** | **G** | **G** | **A** | **C** | **T** |  |  |  |
|  | — | — | — | — | — | — | | — | — | — | — | — | — | — | 2 | 2 |  |
|  | — | — | — | — | — | T | | — | — | A | — | — | — | — | 0 | 2 |  |
|  | — | — | — | — | — | — | | A | — | — | A | — | — | — | 1 | 3 |  |
|  | — | — | — | — | — | — | | A | A | — | A | — | — | — | 1 | 0 |  |
|  | C | — | A | C | — | T | | — | — | A | — | — | — | — | 1 | 1 |  |
|  | C | — | A | C | — | — | | — | — | A |  | G | A | — | 0 | 1 |  |
|  | — | — | A | C | — | T | | — | — | — | — | — | — | — | 1 | 0 |  |
|  | — | — | — | C | — | — | | — | — | — | — | — | — | — | 0 | 2 |  |
|  | — | — | — | C | — | — | | — | — | — | — | — | — | C | 3 | 0 |  |
|  | — | T | — | C | — | — | | — | — | — | — | — | — | — | 0 | 1 |  |

aThe reference HPV16/31/33/52/58 E6/E7gene sequence was NC_001526, J04353.1, M12732.1, NC_001592.1, D90400. b HSIL group, c LSIL and IF group.* Fisher's exact test P value.
